# Antibacterial effect of boron nitride flakes with controlled orientation in polymer composites

**DOI:** 10.1039/c9ra06773f

**Published:** 2019-10-17

**Authors:** Santosh Pandit, Karolina Gaska, V. R. S. S. Mokkapati, Sven Forsberg, Magnus Svensson, Roland Kádár, Ivan Mijakovic

**Affiliations:** Division of Systems Biology, Department of Biology and Biological Engineering, Chalmers University of Technology Kemivagen 10 Goteborg Sweden ivan.mijakovic@chalmers.se; Chalmers University of Technology, Industrial and Materials Science SE 412 96 Gothenburg Sweden roland.kadar@chalmers.se; 2D Fab Bultgatan 20 Sundsvall Sweden; WellSpect Healthcare Aminogatan 1 Goteborg Sweden; Novo Nordisk Foundation, Center for Biosustainability, Technical University of Denmark Kongens Lyngby Denmark

## Abstract

Boron nitride (BN) is a stable 2D material with physiochemical properties similar to graphene-based nanomaterials. We have recently demonstrated that vertically aligned coatings of graphene-based nanomaterials provide strong antibacterial effects on various surfaces. Here we investigated whether BN, a nanomaterial with extensive similarities to graphene, might exhibit similar antibacterial properties. To test this, we developed a novel composite material using BN and low density polyethylene (LDPE) polymer. The composite was extruded under controlled melt flow conditions leading to highly structured morphology, with BN oriented in the extrusion flow direction. Nanocomposite extruded surfaces perpendicular to the flow direction were etched, thus exposing BN nanoparticles embedded in the matrix. The antimicrobial activity of extruded samples was evaluated against *Escherichia coli*, *Pseudomonas aeruginosa*, *Staphylococcus epidermidis* and *Staphylococcus aureus* by the colony forming units (CFUs) counting method. Furthermore, the bactericidal effect of oriented BN against *E. coli* and *S. aureus* was evaluated by scanning electron microscopy (SEM) and live/dead viability assay. Our results suggest that BN nanoflakes on the extruded BN/LDPE composite physically interact with the bacterial cellular envelope, leading to irreparable physical damage. Therefore, we propose that BN–polymer composites might be useful to develop polymer based biomedical devices protected against bacterial adhesion, and thus minimize device associated infections.

## Abstract 2

Vertically oriented boron nitride flakes on a polymer surface exhibit bactericidal activity.

## Graphical abstract

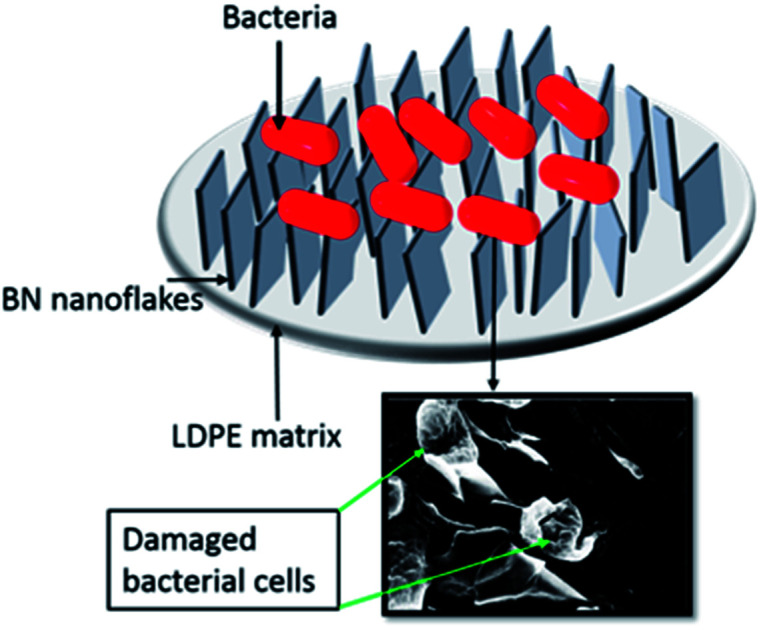

## 1. Introduction

Various advantageous properties of 2D materials have promoted their use in a wide range of applications in biomedical science.^1,2^ One of these materials, graphene, displays unique physiochemical properties such as high surface area, strong mechanical strength, thermal and electrical conductivity and ease of functionalization. This combination of properties made graphene extremely amenable for biomedical applications.^1^ In particular, graphene and its derivatives have been proposed for use in antimicrobial coatings of biomedical implants, biosensors, bioimaging, drug delivery and photothermal therapy.^2–6^ The biocompatibility of graphene-based materials has also been extensively studied. The available results suggested that biocompatibility depends on multiple factors, including size, dose and time of exposure.^5,7,8^ Previous studies have reported that graphene nanoflakes disrupt bacterial cells by penetration into their membranes, and causing complete disintegration.^9^ Cytotoxicity of graphene to mammalian cells can be mitigated by surrounding them with proteins.^10^

Carbon based nanomaterials including carbon nanotubes, graphene, graphene oxide and reduced graphene oxide have been extensively studied for their antibacterial activity.^11–14^ Graphene and graphene oxide nanoflakes have been shown to have strong antibacterial activity against both Gram positive and Gram negative bacteria. The mechanism behind this activity is a direct interaction of nanoflakes with bacterial cell membrane, resulting in physical damage and disintegration of cells.^2,15^ This mechanism has been proven by both experimental and theoretical studies, suggesting that graphene nanoflakes destructively extract phospholipids from bacterial cell membranes and release the intracellular material.^2,15^ In addition to physical damage, generation of oxidative stress by graphene materials to bacterial cells has also been considered as one of the mechanisms for antibacterial activity.^15^ In our previous report, generation of oxidative stress was not observed.^2^

Boron nitride (BN) exhibits a honeycomb structure similar to graphene, with alternating boron and nitrogen atoms. The structure consists of strong sp^2^ covalent in-plane bonding and weak van der Waals forces between layers.^16^ BN nanomaterials are already used in optoelectronic nanodevices, multifunctional composite materials, hydrogen accumulators and insulating substrates.^17^ Considering the structural analogy with graphene-based materials, BN and its derivatives are considered to hold a considerable potential for biomedical applications. Recent studies revealed a remarkable ability of BN to remove oily substances (*e.g.* organics solvent and dyes) from water, as well as applicability in anticancer drug delivery and DNA/RNA self-assembly.^18–20^ Even though there are several proposed applications of BN and its derivatives in the biomedical field, there are no experimental reports on its toxicity/biocompatibility. A previous theoretical study based on molecular dynamics simulations showed the interaction of BN nanoflakes with model cell membranes and demonstrated the spontaneous attraction of BN nanoflakes to the polar head groups of bilayer lipids.^21^

Several *in vitro* and *in vivo* studies carried out with carbon-based materials demonstrated the dose-, time-, and shape-dependent toxicity towards living cells, including human ones.^8,22,23^ Interestingly, BN and its derivatives, such as BN nanotubes, seem to be less toxic and more biocompatible than graphene based materials.^24–26^ The interaction of BN nanotubes with different cells revealed very low levels of cytotoxicity.^24,27,28^ Moreover, BN nanotube films stimulated proliferation of human mammary cells, and plasma treatment of BN nanotube films enhanced cell attachment,^29,30^ suggesting that these materials could hold a considerable potential for various implant technologies. Recent reports on the interaction of BN derivatives (nanoflakes and nanoparticles) with mammalian cells suggest that their biocompatibility depends on their shape, size, and structure.^31^ Despite these promising findings, the use of BN-based materials in biomedical research is lagging far behind their carbon-based counterparts. There are very few reports of assessing antibacterial effects of BN and hybrid BN materials. Recently, a strong bacteriostatic effect of BN–polymer composites against *Escherichia coli* and *Staphylococcus aureus* has been reported, especially with polyethyleneimine coated boron nitride nanotubes.^32^ BN nanomaterials have also been used in the development of electrospun hybrid nanostructure such as BN/Ag and BN/Ag–TiO_2_ hybrid nanomaterials for antimicrobial applications.^33^

By considering the structural similarity of BN with graphene and our previous discovery that vertical alignment of graphene on surfaces leads to the best antibacterial effects,^2,16^ here we developed low density polyethylene (LDPE) composites featuring vertically aligned BN flakes on the surface, in different BN concentrations ranging from 0–20% w/w. Our goal was to investigate the possibility of using these composites for antibacterial applications on polymer based biomedical devices, to prevent associated infections. For this purpose, an industrially compatible process of extrusion has been employed. BN–LDPE composites were extruded in controlled melt flow conditions leading to highly structured morphology, with BN oriented in the extrusion flow direction. Once extruded, BN concentration dependent distribution of BN flakes could be observed on the surface of the extruded and thereafter etched samples. The antimicrobial activity of BN–LDPE composites was evaluated against pathogenic *Escherichia coli*, *Pseudomonas aeruginosa*, *Streptococcus epidermidis* and *Staphylococcus aureus*. The results suggested that BN nanoflakes on the extruded BN–LDPE composite interact with the bacterial cell membrane as expected, leading to cell damage and rupture. Our BN–polymer composites were clearly bactericidal and can therefore be considered for developing polymer based biomedical devices that would reduce device-associated infections.

## 2. Experimental

### 2.1 Preparation of BN–LDPE composites

In this study BN micropowder purchased from Graphene Supermarket with purity of 98% was used. The details regarding average diameter of flakes and their surface area is presented in Table 1. Low density polypropylene (LDPE) was delivered by Borealis AB and it was previously characterized with the use of Gel Permeation Chromatography and Differential Scanning Calorimetry (Table 1).

**Table 1:** Materials parameters

BN		LDPE	
*d*_avg_ [μm]	5	*M*_w_ [kg mol^−1^]	92
*A* [m^2^]	7.5	*M*_w_/*M*_n_	7.6
*ρ* [g cm^−3^]	2.1	*T*_m_/*T*_c_ [°C]	111/94

**Fig. 1:**
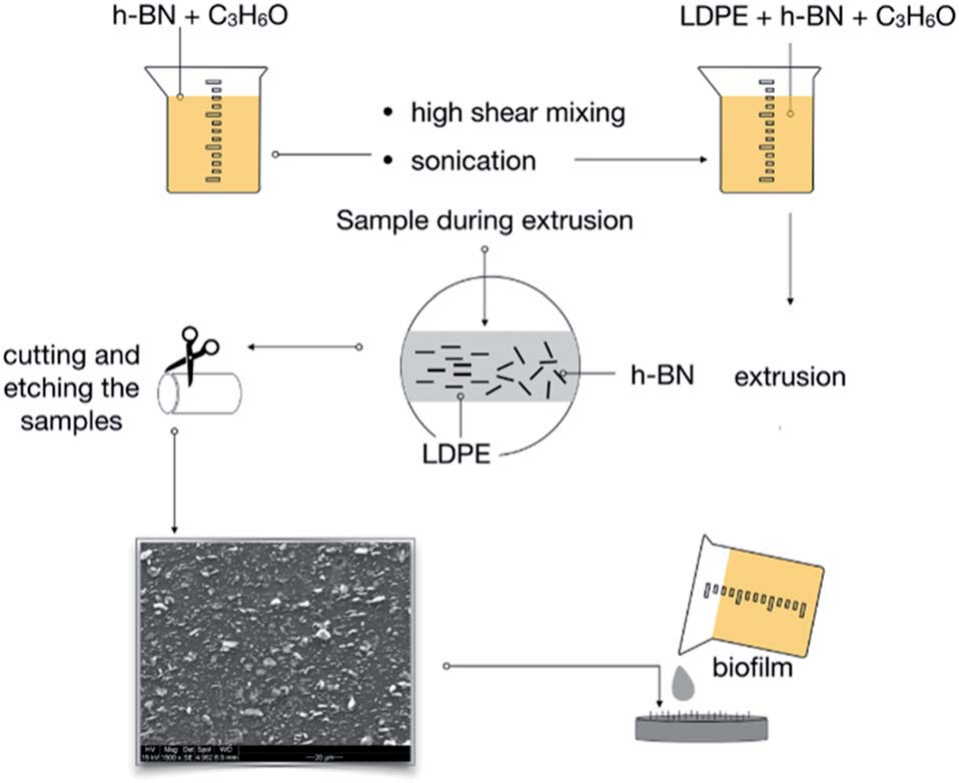
The scheme of preparation and extrusion of BN–LDPE composites with the vertically oriented BN nanoflakes on the surface.

The manufacturing process of BN–LDPE composites is presented in Fig. 1. In the first step, LDPE pellets were cryogenically ground into a powder with average particle size is 0.5 mm, using a high-speed rotor mill. BN micropowder was dispersed in acetone and then sonicated for 3 h, at 90 W. Thereafter, a pre-coating technique was applied as described previously.^34,35^ Briefly, BN-acetone suspension was stirred for 20 min using a rotor-stator mixer Ultra turrax T 25 IKA at 15 000 rpm. In the next step, LDPE powder was mixed with BN-acetone dispersion, using an overhead stirrer rotating for 40 min at 500 rpm until full evaporation of acetone. Master batches with different BN concentrations obtained by this procedure were dried in an oven at 60 °C for 24 h.

### 2.2 Extrusion of BN–LDPE composites

The technique comprised the extrusion of BN polymer nanocomposites using a circular die (Hagen–Poiseuille flow) at sufficiently high shear rates to obtain the orientation of the BN flakes. The input parameters were applied based on the work of Induchoodan *et al.* and Gaska *et al.*^34,36^ LDPE–BN batches were extruded 3 times by means of Brabender 19/25 D (Duisburg, Germany).

Single-screw extruder (compression screw with diameter *D* = 19 mm and screw length of 25*D*, compression ratio 2 : 1) was used. The first 2 extrusions were treated here as melt compounding of the LDPE–BN batches, after which the obtained material was shaped into the final form, namely a cylindrical shape with diameter of 7 mm. The used temperatures, from the compaction zone, melting and metering zones to the extruder's die, were respectively as follows: 115, 130, and 140 °C and a constant speed of 30 rpm was kept during the process. The produced samples varied in filler concentration as follows: 5, 10, 15 and 20% w/w.

### 2.3 Surface treatment

In order to expose the BN flakes, chemical etching was employed. All the samples were etched for 30 h using a solution of 1% w/w potassium permanganate in a mixture of 98% of sulfuric acid, 85% of *ortho*-phosphoric acid and water.^37^ The process was terminated by cleaning the samples in a mixture of sulfuric acid and deionized water, thereafter in hydrogen peroxide and next in deionized water.

### 2.4 Antibacterial ability evaluation

Antibacterial activity of the extruded BN–LDPE composite materials with different BN concentrations was evaluated against *E. coli, P. aeruginosa*, *S. epidermidis* and *S. aureus*. *E. coli* and *P. aeruginosa* was grown in Luria–Bertani (LB) medium, whereas, *S. epidermidis* and *S. aureus* was grown in tryptic soy broth (TSB) at 37 °C, in a shaking incubator. The overnight grown bacterial culture suspensions were diluted to obtain final inoculum of 2–5 × 10^6^ CFU ml^−1^. The diluted bacterial culture suspension was applied on the surface of the BN–LDPE and incubated at 37 °C for 24 h, to allow for biofilm formation. After 24 h of incubation, biofilms were resuspended in 5 ml of 0.89% of NaCl and homogenized by sonication. The homogenized biofilm suspension (100 μl) was serially diluted and plated on LB agar plates, which were then incubated overnight at 37 °C. Thereafter, the number of colonies was counted and the total number of colony forming unit (CFUs) in the original suspension (5 ml of 0.89% NaCl) was calculated to determine the viability of bacterial cells. To visualize the live and dead bacterial cells, control biofilms and biofilms grown on BN–LDPE composite samples were stained for 20 min with the mixture of 6.0 μM SYTO 9 and 30 μM potassium iodide from Live/Dead BacLight Viability kit L13152, (Invitrogen, Molecular Probes, Inc. Eugene, OR, USA). Fluorescence microscopic imaging of the biofilms was performed using a Zeiss fluorescence microscope (Axio Imager.Z2m Carl Zeiss, Jena, Germany). Scanning electron microscopy (SEM) analysis of biofilms was performed as described previously.^38^ Briefly, biofilms were fixed with 3% of glutaraldehyde solution for 2 h and dehydrated with graded series of ethanol concentrations (40, 50, 60, 70, 80, 90%) for 15 min each and with absolute ethanol for 20 min. The dehydrated biofilm samples were dried overnight at room temperature and coated with a thin layer of gold before SEM imaging. SEM imaging was performed with Supra 55 VP (Carl Zeiss AG Jena, Germany).

### 2.5 Statistical analysis

The data is presented as mean ± standard deviation from three biological replicates. Intergroup differences were estimated by one-way analysis of variance (ANOVA), followed by a post hoc multiple comparison (Tukey) test to compare multiple means. Values were considered statistically significant when the *P* value was <0.05.

## 3. Results and discussion

**Fig. 2:**
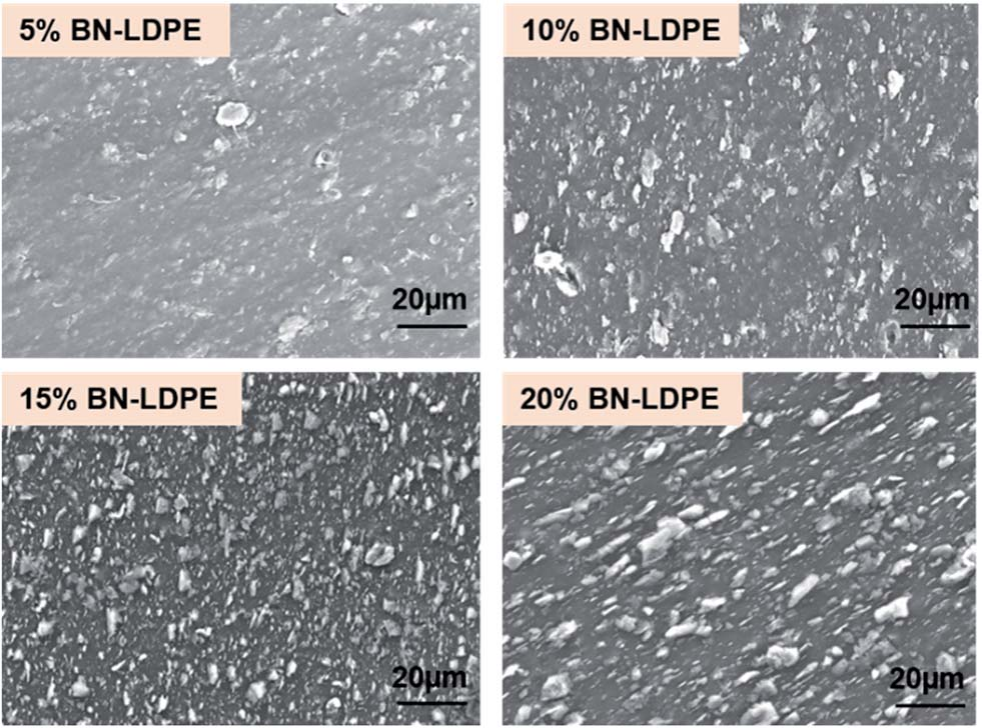
SEM images showing the vertical orientation of BN on the extruded BN–LDPE composites with 5–20% of BN.

The BN–LDPE composite was extruded with controlled shear force to generate the orientation of BN nanomaterials on the surface (Fig. 1). The longitudinal section of extruded composite materials was etched to expose the alignment of BN nanomaterials on the top surface. Fig. 2 shows the alignment of BN on the top surface of BN–LDPE composites with different concentrations of BN. It can be clearly observed that BN is tightly stacked with LDPE and nanoflakes are vertically oriented (perpendicular to the surface). Furthermore, the density of oriented nanoflakes was observed to be concentration dependent. Both extensional and shear flow can be responsible for filler orientation. However, in this extruder configuration, the orientation is induced through the shear Poiseuille flow inside the die.^36^ In the case of a Poiseuille flow through a tube, nanofillers are oriented such that their principal axes are along the flow direction and along concentric iso-velocity planes. In our samples, BN nanoflakes are oriented in the polymer flow direction.

There are several available studies demonstrating multiple antibacterial mechanisms of graphene materials, including piercing the cellular envelope with sharp exposed edges, oxidative stress and wrapping of the bacterial cells leading to death caused by preventing transmembrane transport of nutrients.^15,39,40^ Piercing of bacterial cells by exposed sharp edges of nanoflakes, leading to damage of bacterial cells, is widely accepted as a major antibacterial mechanism. It has been demonstrated that the direct contact of bacterial cells to sharp edges of graphene nanoflakes results in loss of bacterial membrane integrity and leakage of intracellular content.^2^ Furthermore, these sharp edges were also demonstrated to induce significant membrane stress on bacteria *via* the “chopping” effect.^15,39^ It is clear from the previous studies that sharpness and orientation of nanomaterials are an important parameter to achieve significant bactericidal effects. The coatings of antibacterial materials such as graphene oxide and metallic nanoparticles have been reported to have notable bactericidal effects.^11,41^ Most of the nanomaterials having strong antibacterial activity are also shown to have toxicity towards the mammalian cells, in a dose- and time-dependent manner.^42^ By contrast, BN nanomaterials were reported as more biocompatible, *i.e.* exhibiting less toxicity than carbon nanomaterials.^24,27,28^ However, the detachment and release of flakes or similar structures from coated devices might lead to toxicity towards human cells. In order to avoid this effect, the used nanomaterials should be strongly integrated with the substrate to prevent their release to outer environment. Therefore, incorporation of nanoflakes in a polymer matrix seems a viable route to preventing the release of BN nanomaterials. As shown in Fig. 2, the boron nitride nanoflakes are stably embedded withing the polymer, with no observable nanomaterial detachment from the composites.

**Fig. 3:**
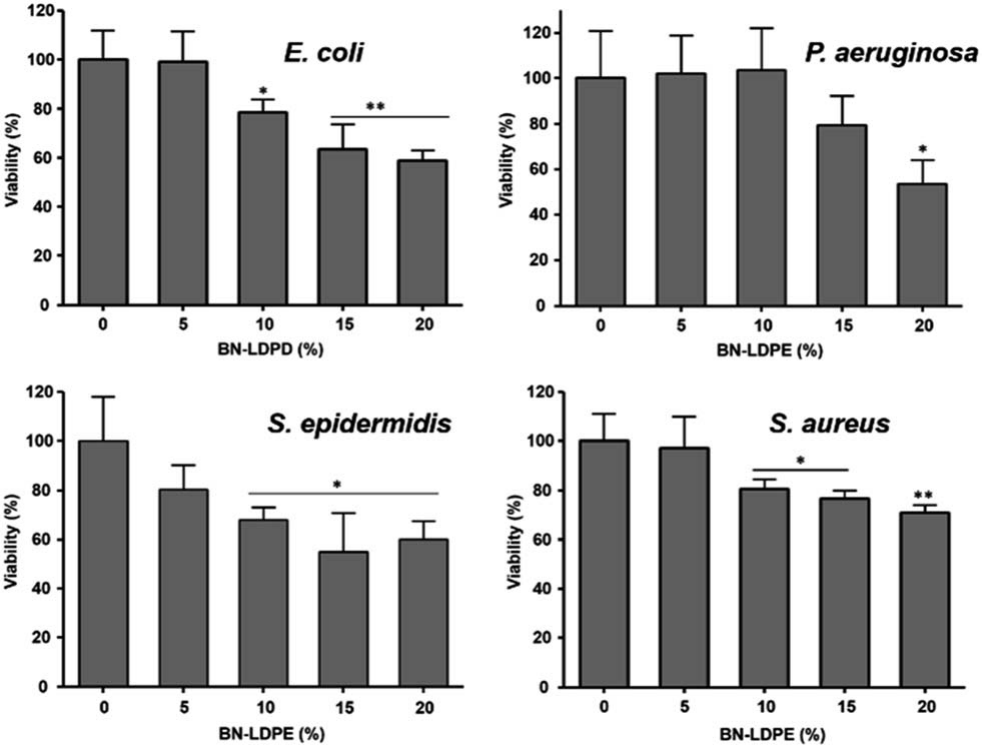
Measurement of bactericidal activity extruded LDPE composites with 0–20% of BN after the 24 h of bacterial growth. Results are presented as a mean ± standard deviation error of viability percentage of each bacterial strains in compared to control. *P < 0.005, **P < 0.001.

Antibacterial activity of BN–LDPE composites was tested by culturing bacterial biofilms on these samples, using some common opportunistic pathogens. The viable bacterial cells from the biofilm were counted by plating the biofilm suspension. The morphology of bacterial cells on the biofilm was examined by scanning electron microscopy and the viability of bacterial cells on biofilm was examined by live/dead staining. As shown in Fig. 3, for all bacterial strains the viability decreased proportionally to the increase in BN concentration in the composite materials. The bactericidal activity of the composite was in accordance with the density of BN nanoflakes on the surface, which also correlated with the content of BN, Fig. 2. At BN concentration in the composites of ≥10%, there was significant loss of viability of *E. coli*, *S. aureus* and *S. epidermidis* compared to the pure LDPE control surface. The BN–LDPE composite seemed to be less effective against *P. aeruginosa* compared to other bacterial strains, showing significant decrease in viability only with 20% of BN in the composite. The reason behind increased resistance of *P. aeruginosa* was not further investigated in this study. Nevertheless, we presume that it could be related to comparatively large amounts of exopolymeric substances (EPS) produced by *P. aeruginosa*, which might prevent direct contact between BN nanoflakes and bacterial cells.

**Fig. 4:**
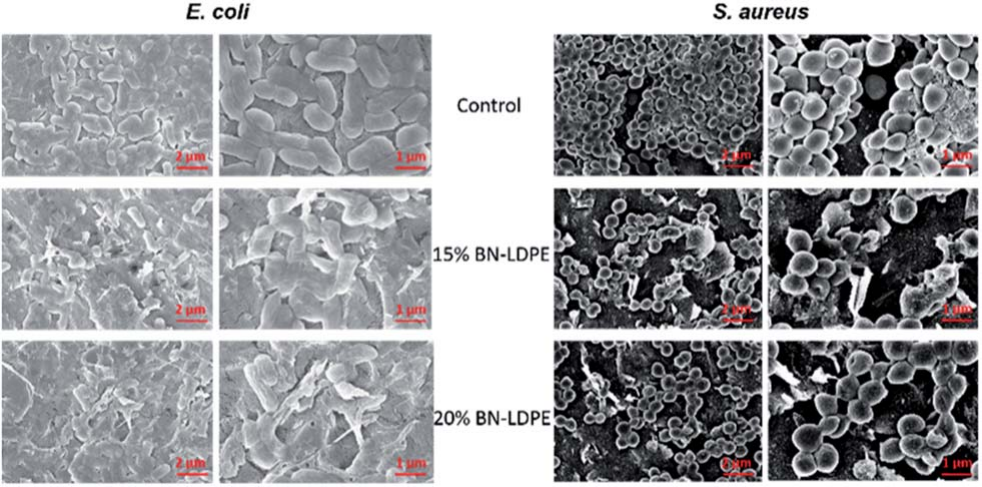
Representative SEM images of *E. coli* and *S. aureus*. *E. coli* and *S. aureus* were grown for 24 h on LDPE (control) and LDPE composites with 15 and 20% of BN–LDPE composites and grown bacterial cells were fixed and dehydrated before SEM imaging.

Fewer viable cells on BN–LDPE composite compared to BN samples might be due to either an inhibitory effect of BN nanospikes on the adhesion of bacteria or could be caused by direct bactericidal activity, *i.e.* killing by BN nanoflakes. In order to assess this, we analyzed the live/dead ratio of bacterial cells grown on LDPE and BN–LDPE composites. Biofilms grown on these samples were stained with Syto 9 and propidium iodide and examined under a fluorescence microscope. Propidium iodide permeates and stains specifically the dead cells (red), while the living cells are not stained (green). The results are shown in Fig. 4. After 24 h of incubation, a large number of dead cells (red) was observed on composites with 15 and 20% of BN, compared to the LDPE control. Results obtained from this experiment were fully in correlation with the findings from the CFU counting method and suggested that BN does not have a major effect on bacterial attachment, but rather acts by killing the bacteria directly.

**Fig. 5:**
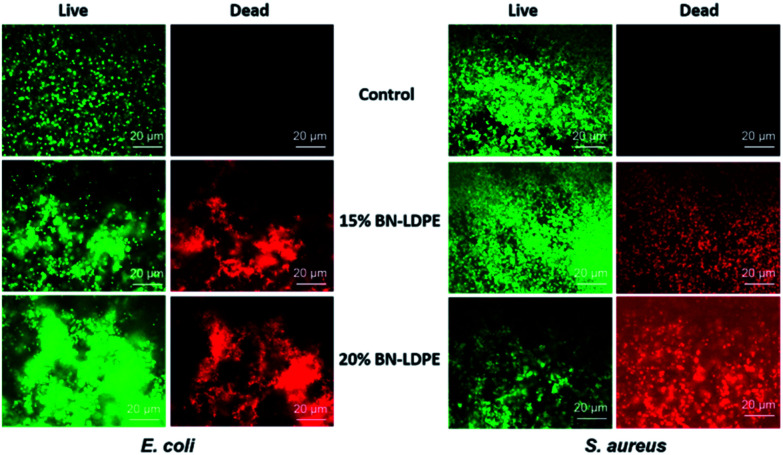
Representative fluorescence microscopic images of *E. coli* and *S. aureus*. *E. coli* and *S. aureus* were grown for 24 h on LDPE (control) and LDPE composites with 15 and 20% of BN–LDPE composites and stained with live/dead bacterial viability kit and images were acquired by using fluorescence microscope. Green color represents the live bacterial cells and red color represents the dead bacterial cells.

The exact molecular and physical mechanism behind the bactericidal effect of BN has not been fully explained. The bacterial cells grown on LDPE composite without BN showed a normal healthy morphology (Fig. 5), which is consistent with LDPE being fully bio-compatible. By contrast, the morphology of bacterial cells grown on BN–LDPE composite was affected, suggesting substantial damage to the cell envelope (Fig. 5). Morphology of the Gram-negative *E. coli* grown on BN–LDPE was flattened, whereas the Gram-positive *S. aureus* cells were squashed and wrinkled but not completely flattened. The difference in the level of morphological disruption in between these bacterial cells might be due to the difference in the cell wall composition. Most of the Gram-positive bacteria have a thick cell walls with thick layer of peptidoglycan (around 20–80 nm) which requires more physical/chemical stress to completely distort, whereas Gram-negative bacteria, having thinner peptidoglycan layer (around 5–10 nm), are comparatively more prone to distortion under physical/chemical stress.^43^ For both species, there are many cells with visible physical damage of the envelope, suggesting that BN nanomaterials have a similar antibacterial mechanism as previously reported vertical graphene coatings.^2^

## 4. Conclusion

In this study we report a facile strategy for the production of composites with bactericidal activity that can be used to produce polymer-based biomedical devices. Bactericidal activity of BN–LDPE is clearly demonstrated, and it is proportional to the concentration of BN. According to SEM examination, deformation and lysis of bacterial cells occur when the bacterial cells in the biofilm come in contact with the sharp edges of BN nanoflakes. The density of nanoflakes on the composite samples directly correlates with the bactericidal efficacy of the surface. While BN materials exhibit similar antibacterial properties as graphene, they hold an advantage in terms of biocompatibility with human cells and tissues. Therefore, BN-enhanced polymeric composites with bactericidal activity might constitute a promising venue for production of intermittent medical devices such as shorter-term catheterization or similar procedures.

## Conflicts of interest

The authors declare no conflict of interest.

## Supplementary Material
